# How to Feed Cleft Patient?

**DOI:** 10.5005/jp-journals-10005-1198

**Published:** 2013-08-26

**Authors:** Mahendra Kumar Jindal, Saima Yunus Khan

**Affiliations:** Associate Professor, Department of Pedodontics, Dr ZA Dental College, Aligarh, Uttar Pradesh, India, e-mail: dct_mkj@yahoo.co.in; Assistant Professor, Department of Pedodontics, Dr ZA Dental College, Aligarh, Uttar Pradesh, India

**Keywords:** Cleft lip and palate, Infancy, Feeding

## Abstract

Cleft lip and palate patients have all rights like other normal individuals, to enjoy the benefits of nourishment. Knowledge has to be there about the different feeding positions like straddle, dancer hand position along with the use of specially designed bottles and nipples. Parent's should be trained about the correct positions of feeding, in extreme of the cases in which parents are not able to follow these instructions, feeding obturators can be given.

**How to cite this article:** Jindal MK, Khan SY. How to Feed Cleft Patient? Int J Clin Pediatr Dent 2013;6(2):100-103.

## INTRODUCTION

The Indian subcontinent remains one of the most populous areas of the world, with a birth prevalence of clefts somewhere between 27,000 and 33,000 clefts/year.

The feeding difficulties associated with cleft palate and lip patients has been well documented for many years, the main underlying problem is failure to generate sufficient negative intraoral pressure during suction, lack of which is associated with the following problems such as nasal regurgitation, excessive air intake, fatigue, choking, exhaustive prolonged feeds, frequent burping and discomfort to the mother.^1^ The immediate concern for the baby is good nutrition, as a number of studies have reported slow weight gain in infants with cleft palate, especially in the first few months of life. This article would like to focus that with some adjustments to feeding methods and positioning, the baby would be able to get all the benefits of feeding, i.e. bonding with the mother, adequate nutrition for growth and development^2^ before undergoing with surgery either for the lip or the palate.

## FEEDING METHODS

Breast, bottle, spoon or cup feeding or a combination may be chosen with lactation instruction pertaining to the correct positioning of the child. Even though breast feeding may be difficult for a baby with cleft palate, there is an option for the mother to express her milk and give it through the bottle. We would strongly like to advocate the importance of breast milk over formula milk, breast milk with mother's antibodies helps to combat infection, is less irritating to mucous membrane, is available at body temperature and does not need any prior sterilization.^3^ Depending on the anatomical defects, we recommend the following methods of feeding:

### Babies with Cleft Lip

**Fig. 1:**
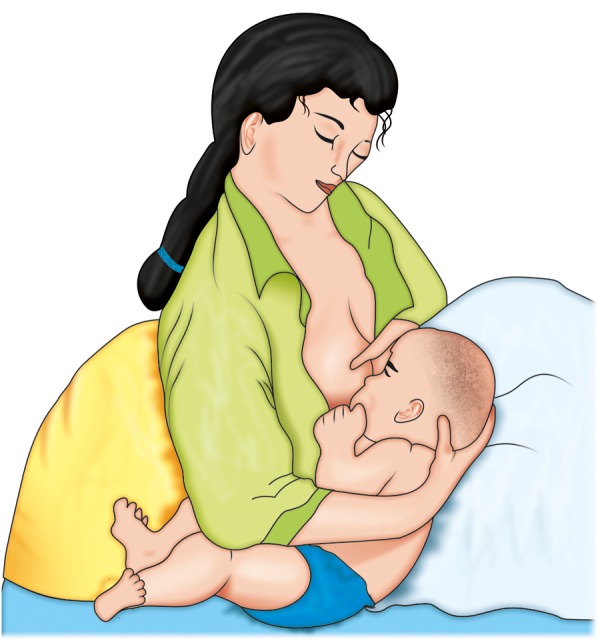
Modified football method or straddle position for feeding

**Fig. 2:**
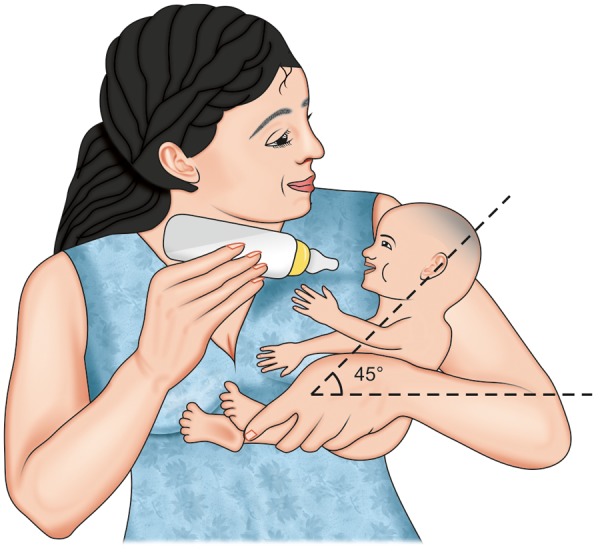
Correct position of feeding

**Fig. 3:**
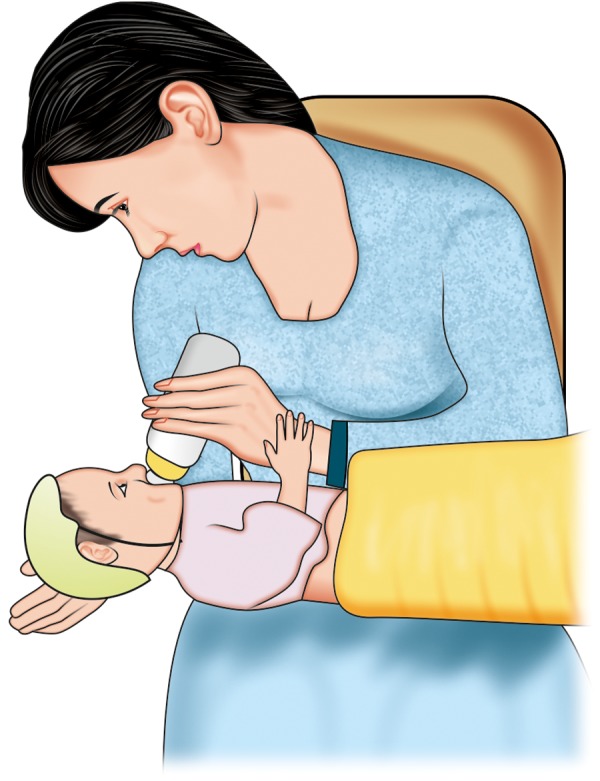
Incorrect position of feeding

Do not have a major problem with feeding, need some modifications in positioning during feed. If cleft is unilateral, use of modified football method or straddle position (Fig. 1) may be helpful. In this method position the baby with cleft toward the breast, this allows the cleft to be tucked into the breast tissue and makes it easier for the baby.^4^Further support to infant's cheek, decreases the width of the cleft which simultaneously increase the closure around the nipples. In all feeding positions, baby is kept in an upright position (Fig. 2), this allows the milk to flow down and helps prevent choking. If incorrect position is taken (Fig. 3) milk may enter the respiratory passage.

**Fig. 4:**
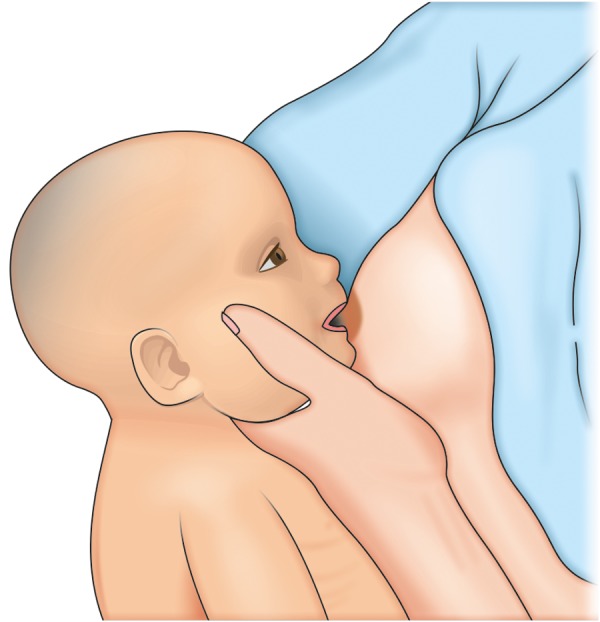
Dancer hand position for feeding

Breast feeding would be more difficult for babies with bilateral cleft of the lip; this is due to the inability to form an airtight seal around the nipple. ‘Dancer hand position’ is recommended (Fig. 4). Slide the hand under the breast forward, i.e. supporting the breast with three fingers rather than four form a U-shape with the thumb and forefinger to cradle the baby's chin, this helps the baby to press the nipple and areola between the gums. In conditions where breast feeding is not adequate, one can always switch on to specially designed bottles, as nutrition cannot be compromised.

### Babies with Cleft of the Soft Palate

He or she may be able to feed from the breast with correct positioning, as discussed above. In some cases, they may need supplementary feeds from specially designed bottles with either expressed breast milk or formula milk.

### Babies with Cleft of the Lip, Soft and Hard Palate

**Fig. 5:**
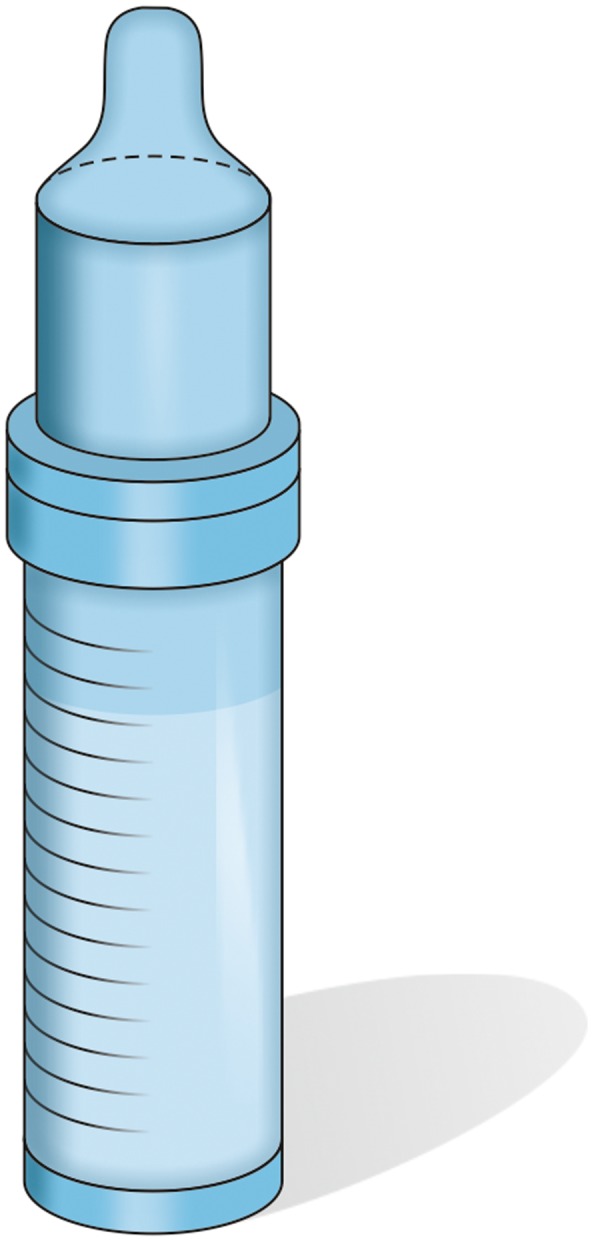
Haberman feeder bottle for feeding

**Fig. 6:**
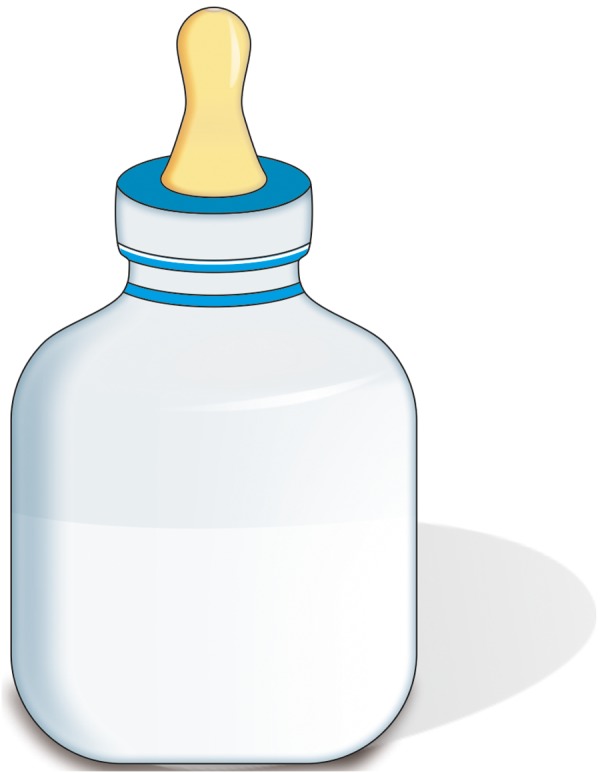
Mead Johnson cleft palate Nurser bottle for feeding

**Fig. 7:**
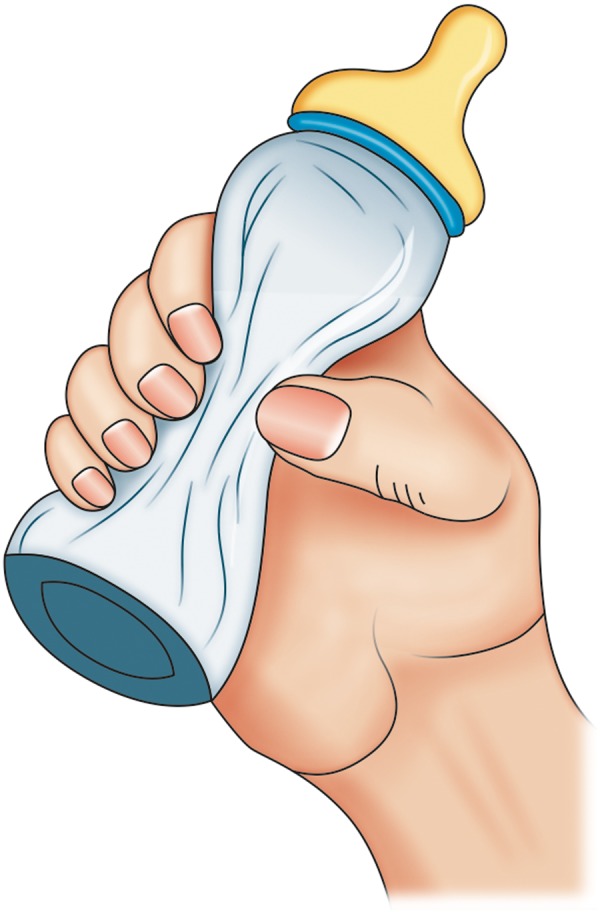
Squeezable bottle for feeding

**Fig. 8:**
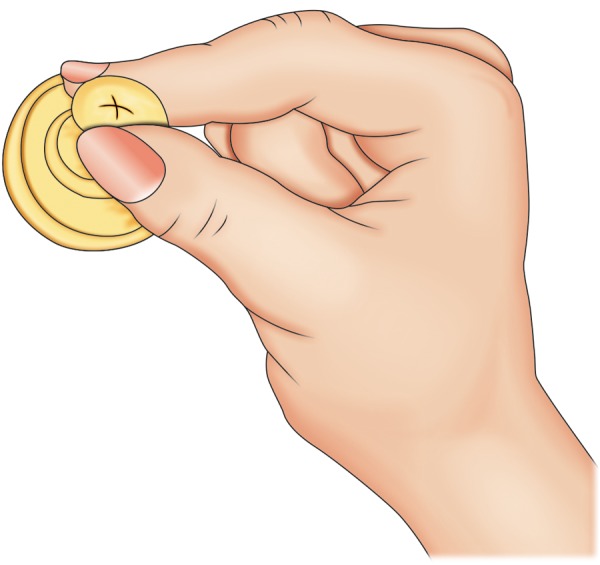
Cross cut nipple for feeding

In most cases, these babies are unable to breast-feed though one can always try to breast-feed. If breast feeding is not achieving the outcomes, then it may be necessary to bottle feed as well. Various specially designed feeding bottles and teats like Haberman feeder (Fig. 5), Mead-Johnson cleft palate nurser bottle (Fig. 6), Pigeon bottle are available. These bottles are made up of soft, squeezable plastic to help draw milk from the bottle with very little pressure (Fig. 7). A long nipple to press against the tongue, with a Y-cut in the tip of the nipple (Fig. 8) is recommended.^5^ Position has to be as upright as possible, with the head in one hand and the bottle in another. With these squeeze bottles, it is good to practice first with water, so as to determine how firmly and frequently the bottle needs to be squeezed to get a steady flow.

## SPOON AND CUP FEEDING

Before operation to repair cleft palate, baby needs to be completely weaned from bottle drinking, reason being that after palate repair the bottle nipple can rub against the stitches and break down the repair. A long handled spoon with a flat bowl should be used. With spoon feeding, first introduce pabulum and cereals. Introduction of only one ingredient of food at a time should be done.^6^

Some foods may be irritating to the nasal passage such as citrus foods and tomatoes, as they have an acidic quality. Hence, once the child gains more control, eating these types of foods would be easier later on. As regards the cup, there is no specific cup for a child with cleft palate, but the lactation instructions have to be followed. Nasogastric feeding can be done if the baby is in a debilitated condition, otherwise should be avoided.

## PROSTHESIS

Feeding obturators are passive devices designed to provide a normal contour to the patients of cleft alveolus and hard palate. They separate the oral and nasal cavities and provide a surface to oppose the nipple. Obturators have been a major area of controversy. There are two schools of thoughts, the first one advocates its advantages – that they help in feeding, facilitate lip and palate repair, speech and facial growth are enhanced. The other group claims its disadvantages–its impression taking procedure too cumbersome for the infant, second most important criteria is its sterilization and hygiene maintenance. To date there is no evidence to support or disown its use. The practice remains empirical.^7^

To summarize, apart from the proper feeding positions, these following instructions should also be given to the parents:

- Feed often, at least 8 to 12 times in 24 hours.
- Feeding not to be exhaustive, limit to less than 30 minutes.
- Burping more often, 2 to 3 times during feed.
- Oral hygiene maintenance, i.e. gum pad and prosthesis if given.
- Sterilization of feeding bottles, nipples.
- Reassurance with patience as babies with clefts take longer time to feed.
- A protocol should be maintained to examine the growth and development in babies with cleft.

## CONCLUSION

There is certainly lack of knowledge about specially designed squeezable bottles and nipples and the different methods and positions of feeding, not only in the general population but with the clinician's also. It is this lack of knowledge which is having its impact on the market with respect to the nonavailability of these bottles and nipples. Till date no data is available in Indian literature about the clinical efficiency of these methods and eventually the patient is the sufferer. Proper counseling and training should be made available about the correct feeding position. In cases where the parents are not able to follow these instructions, feeding obturators can be advocated along with instructions on its hygiene maintenance. This article is based on our experience with the patients. Though there seems no single intervention that can be prescribed with confidence to improve feeding; more in depth exploration is awaited in this field.
